# An Unexpected Passage: A Complex Enterovesicular Fistula

**DOI:** 10.7759/cureus.4111

**Published:** 2019-02-20

**Authors:** Leon D Averbukh, Faripour A Farouhar, Ethan I Bortniker

**Affiliations:** 1 Internal Medicine, University of Connecticut Health Center, Farmington, USA; 2 Pathology, University of Connecticut Health Center, Farmington, USA; 3 Gastroenterology, University of Connecticut Health Center, Farmington, USA

**Keywords:** enterovesicular fistula, crohn's disease, fecaluria

## Abstract

Enterovesicular fistulas (EVFs) are abnormal connections between the colon and the urinary bladder. They are estimated to account for one in every 3,000 surgical hospital admissions and are rarely associated with long-standing Crohn’s colitis. We present an interesting case of a 93-year-old man with a long-standing history of Crohn’s colitis on mesalamine, whose mechanical fall at home lead to the discovery of a colovesicular fistula with invading urothelium concerning for squamous cell carcinoma.

## Introduction

Enterovesicular fistulas (EVFs) are abnormal connections between the colon and the urinary bladder. They are estimated to account for one in every 3,000 surgical hospital admissions with the most common etiology being diverticulitis (65%-79% of cases), followed by malignancy (10%-20%), and, much less commonly, long-standing Crohn’s colitis (5%-7%) [1]. Clinically, the classic presenting features of EVFs are pneumaturia, fecaluria, and recurrent polymicrobial urinary tract infections [2]. EVFs are most commonly seen in patients in their fifth to seventh decades of life and predominantly in men with a male-to-female ratio of roughly 2-3:1 [3]. In this case report, we examine the admission of a 93-year-old man with a longstanding history of Crohn’s colitis on mesalamine, whose mechanical fall at home lead to the discovery of a colovesicular fistula with invading urothelium concerning for squamous cell carcinoma.

## Case presentation

A 93-year-old male presented to the emergency department (ED) after experiencing a witnessed mechanical fall at home. In the weeks leading up to the fall, the patient had been treated for a urinary tract infection (UTI) with a course of antibiotics. After the initial diagnosis of UTI, the patient experienced over eight episodes of watery, non-bloody diarrhea a day. The patient’s past medical history was significant for longstanding Crohn’s colitis, complete heart block, hypertension, and a seizure disorder. At the time of presentation, the patient was only taking his home medications, which were atorvastatin 40 mg daily, ezetimibe 10 mg daily, lisinopril 40 mg daily, amlodipine 5 mg daily, phenytoin 300 mg daily, acetaminophen 650 mg daily, vitamin B1 100 mg daily, vitamin D3 1000 units daily, and mesalamine 2.4 grams daily.

**Figure 1:**
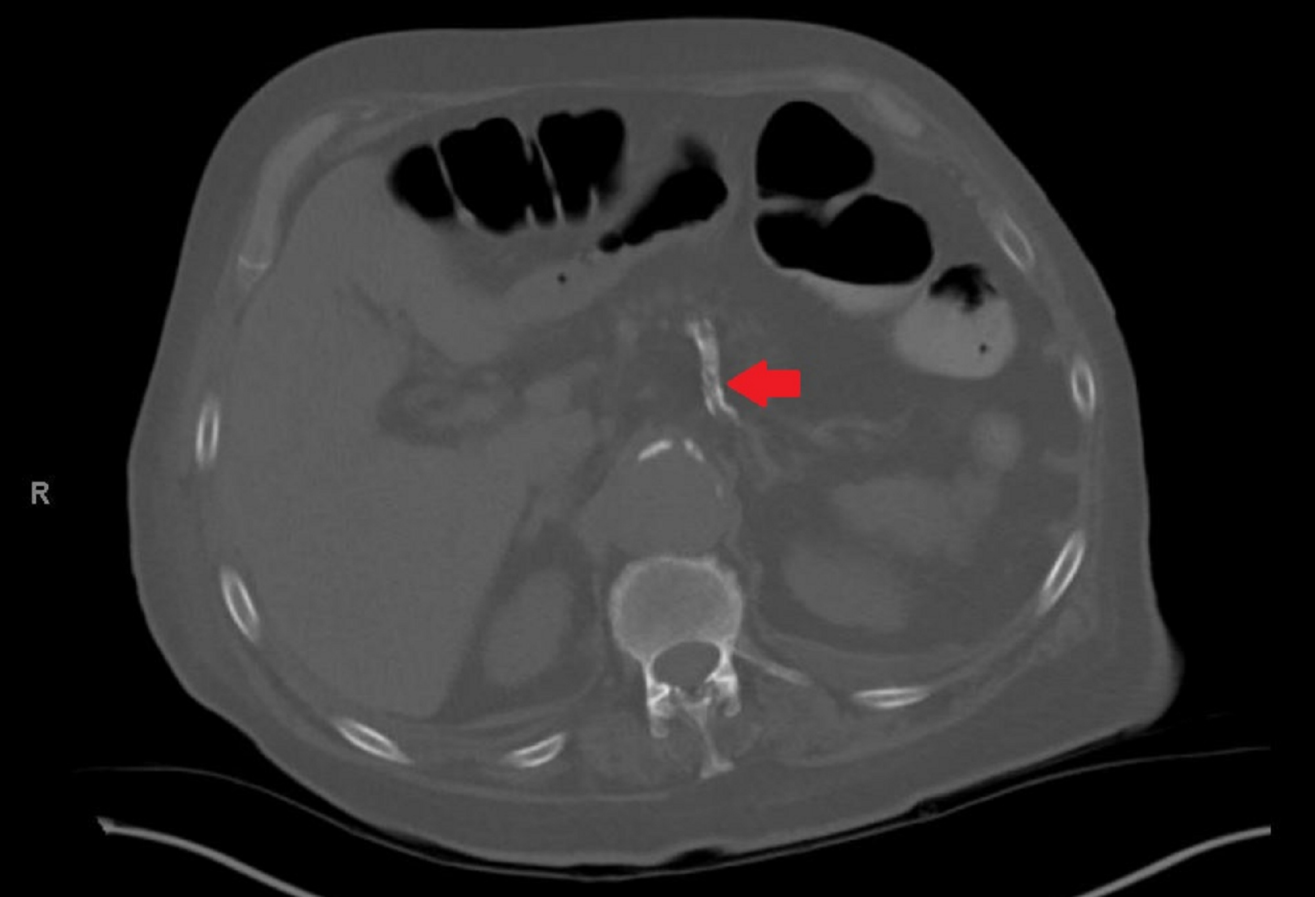
CT abdomen and pelvis with oral and rectal contrast showing fistulation between the sigmoid colon and the bladder (red arrow). CT: computed tomography

**Figure 2:**
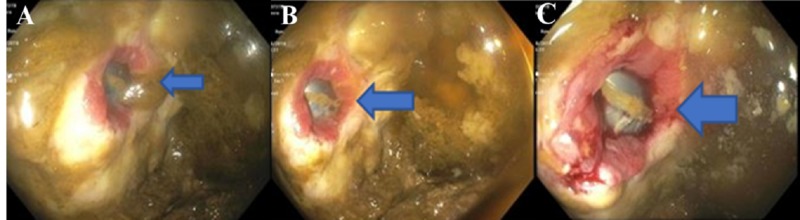
Flexible sigmoidoscopy imaging showing a fistula within the sigmoid colon (blue arrows, panels A, B, and C).

**Figure 3:**
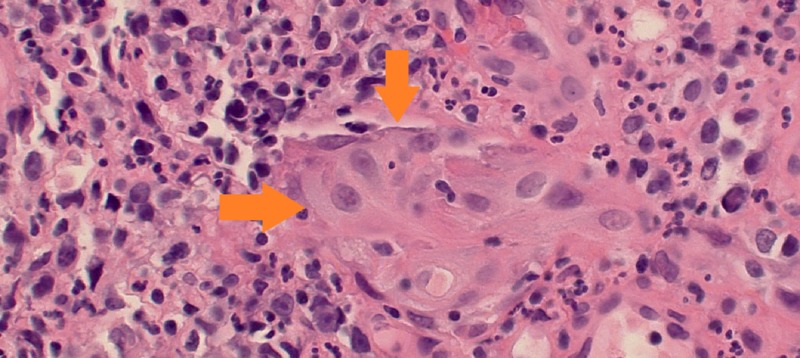
Entrapped urothelium with focal squamous metaplasia within the granulation tissue wall of the fistula tract. Cytologic atypia and the pseudo-invasive pattern raises the question of infiltrating urothelia or squamous carcinoma of bladder (orange arrows).

While in the ED, the patient had a temperature of 100.4 degrees Fahrenheit. Physical exam was significant only for mild suprapubic tenderness. On laboratory studies, the patient had an elevated white blood cell count of 11,200 uL and acute kidney injury (AKI) with a blood urea nitrogen and creatinine of 32 mg/dL and 1.7 mg/dL (baseline creatinine of 1.0 mg/dL), respectively. A Foley catheter was placed and subsequent drainage was significant for feculent material. Urine cultures grew over 100,000 colonies of Escherichia coli, over 1000 colonies of Enterococcus casseliflavus, and over 1000 colonies of Enterococcus enterodurans. The patient’s blood cultures remained negative throughout his hospitalization. He was started on ceftriaxone and metronidazole for his urinary infection and once his AKI improved, he underwent computerized tomography (CT) abdomen and pelvis with oral and rectal contrast. The CT scan showed evidence of a sigmoid EVF with intraluminal gas and diffuse wall thickening of the bladder (Figure 1). Due to concern for fistulization between the sigmoid colon and the bladder, the patient underwent a flexible sigmoidoscopy. The study found an approximately 24 mm fistula opening in the sigmoid colon leading to the bladder (Figure 2). Biopsy of the fistula showed a pyogenic granuloma with urothelial nests concerning for possible squamous carcinoma of the bladder (Figure 3). Due to the patient’s comorbidities, it was decided against surgical intervention for fistula closure or colostomy diversion, and he was discharged once his AKI further improved and his diarrhea ceased.

## Discussion

Our patient’s sigmoid EVF was likely in the setting of long-standing Crohn’s colitis based on the granulomatous tissue found on fistula biopsy. EVFs are uncommon in Crohn’s colitis and emerge due to abscess formation or bowel perforation from the development of aphthous ulcers [4]. Though most fistulas in Crohn’s colitis are found in the perianal area, those patients that do develop EVFs usually have ileovesicular fistulas due to the proximity of the ileum to the bladder dome [4]. Interestingly, our patient’s Crohn’s colitis was reportedly well-controlled on a maintenance dose of mesalamine and he did not have any recent history of Crohn’s flares. Due to his advanced age, he did not have any recent colonoscopies. Chart review revealed no prior documentation of an enterovesicular fistula.

Moreover, our patient’s fistula had a peculiar finding on histology: nests of urothelium with atypia and high mitotic activity. Many of the urothelial nests contained a lumen, suggesting that they were probably submucosal glands of the bladder lined by atypical urothelium. Focal areas of squamous differentiation were also present. It is unclear as to the origin of these cellular nests, as on cystoscopy, the patient did not have any indication of malignancy. A review of available literature showed that vesicocolonic fistula formation secondary to primary bladder malignancy has been previously reported though it is incredibly rare, with only two published cases on the subject [5-6]. In our case, while the finding is highly unusual, it is less likely to be the cause of the fistula and more likely to be cellular seeding and infiltration of unclear origin based on the nesting pattern of the urothelial cells within the fistula.

Operative management is the treatment of choice for EVFs of both malignant and nonmalignant origin. It was previously believed that foregoing operative repair of EVFs predisposes patients to urosepsis or uremia, with up to 75% of affected patients dying from septic complications [7]. However, contemporary studies have reported much lower complication rates, with some fistulas displaying spontaneous closure [8-9]. For those with minimally symptomatic fistulas of nonmalignant nature, expectant management may be the preferred treatment option. In the case of our patient, while his fistula was large and contained cells concerning for malignancy, an informed decision was made against surgical intervention due to his multiple comorbidities as well as overall life expectancy.

## Conclusions

EVFs are a common cause of surgical hospital admissions and are most frequently seen in the setting of diverticulitis, malignancy, and, rarely, in long-standing Crohn’s colitis. While operative management is still considered the treatment of choice for EVFs, previously described complication rates without surgical correction have recently been called into question. In some cases, where the fistula is minimally symptomatic and of nonmalignant nature, expectant management is a treatment option.

## References

[1] Golabek T, Szymanska A, Szopinski T, Bukowczan J, Furmanek M, Powroznik J, Chlosta P (2013). Enterovesical fistulae aetiology, imaging, and management. Gastroenterol Res Pract.

[2] Aiken WD, Reid G, Powell LP (2015). Appearance of a colovesical fistula at cystoscopy. Clin Case Rep.

[3] Smeenk RM, Plaisier PW, van der Hoeven JA, Hesp WL (2012). Outcome of surgery for colovesical and colovaginal fistulas of diverticular origin in 40 patients. J Gastrointest Surg.

[4] Wade G, Zaslau S, Jansen R (2014). A review of urinary fistulae in Crohn's disease. Can J Urol.

[5] Sellers W, Fiorelli R (2015). Enterovesical fistula secondary to squamous cell carcinoma of the bladder. Urol Case Rep.

[6] Yang CH, Liu KH, Chen TC (2009). Enterovesical fistula caused by a bladder squamous cell carcinoma. World J Gastroenterol.

[7] Garcea G, Majid I, Sutton CD, Pattenden CJ, Thomas WM (2006). Diagnosis and management of colovesical fistulae; six-year experience of 90 consecutive cases. Colorectal Dis.

[8] Amin M, Nallinger R, Polk Jr HC (1984). Conservative treatment of selected patients with colovesical fistula due to diverticulitis. Surg Gynecol Obstet.

[9] Solkar MH, Forshaw MJ, Sankararajah D, Stewart M, Parker MC (2005). Colovesical fistula - is a surgical approach always justified?. Colorectal Dis.

